# Decadal epidemiology of malaria in KwaZulu-Natal, a province in South Africa targeting elimination

**DOI:** 10.1186/s12936-019-3001-x

**Published:** 2019-11-20

**Authors:** Rajendra Maharaj, Ishen Seocharan, Bheki Qwabe, Moses Mkhabela, Sunitha Kissoon, Vishan Lakan

**Affiliations:** 1 Office of Malaria Research, South African Medical Research Council, Durban, South Africa; 2 School of Life Sciences, College of Agriculture, Engineering and Science, University of KwaZulu-Natal, Pietermaritzburg, South Africa; 3 School of Public Health and Surveillance, Faculty of Science, University of Pretoria, Pretoria, South Africa; 4 Biostatistics Research Unit, South African Medical Research Council, Durban, South Africa; 5 KwaZulu-Natal Department of Health, Jozini, South Africa

**Keywords:** Decadal, Malaria elimination, Imported malaria, Indoor residual spraying

## Abstract

**Background:**

Although malaria remains a noteworthy disease in South Africa, the provinces are at differing stages of the malaria elimination continuum. KwaZulu-Natal has consistently reported the lowest number of cases over the past 5 years and it is expected that the goal of elimination will be achieved in this province over the next few years. The study reports on few key indicators that realistically represents the provinces progress over the past decade. Local and imported morbidity and mortality is seen as the key indicator as is malaria in children under the age of five and pregnant women. The only vector control intervention in the province is indoor residual spraying (IRS) and this gives an estimate of the population protected by this intervention.

**Methods:**

Trend analysis was used to examine the changing epidemiology in KwaZulu-Natal over the past decade from 2008 to 2018. The data used in this decadal analysis was obtained from the provincial Department of Health. Since malaria is a medically notifiable disease, all malaria cases diagnosed in the province are reported from health facilities and are captured in the malaria information system in the province.

**Results:**

The results have shown that imported cases are on the increase whilst local cases are decreasing, in keeping with an elimination objective. Preventing secondary cases is the key to reaching elimination. Only 10% of the cases reported occur in children under 5 years whereas the cases in pregnant women account for about 1% of the reported cases. Over 85% of the houses receive IRS and this is also the same proportion of the population protected by the intervention.

**Conclusion:**

Several challenges to elimination have been identified but these are not insurmountable. Although there are major impediments to achieving elimination, the changing epidemiology suggests that major strides have been made in the past 10 years and KwaZulu-Natal is on track to achieving this milestone in the next few years.

## Background

According to the 2018 World Malaria Report [1], malaria accounts for the death of more than 435,000 people and causes illness in around 219 million people annually. The majority of these cases and deaths happen on the African continent. Malaria cases in South Africa increased dramatically from 2016 resulting in the highest number of cases being reported post-2000 epidemic in 2017. The northern and north-eastern border regions of South Africa are the primary areas for malaria transmission in South Africa [2]. The provinces of Limpopo, Mpumalanga and KwaZulu-Natal are located along these border regions. Extending for 800 km along the east shoreline of South Africa, KwaZulu-Natal is partitioned into eight districts. uMkhanyakude District in northern KwaZulu-Natal is one of the 8 districts and located within this district is the Jozini Local Municipality. Jozini Local Municipality is one of the districts in South Africa that experiences malaria transmission. Malaria is a disease of poverty particularly among impoverished, rural groups that have constrained access to health care services and reside in homes that offer few barriers against mosquitoes [3]. KwaZulu-Natal borders the neighbouring countries of eSwatini and Mozambique that contributes an estimated 77% of malaria cases annually [4].

The predominant malaria parasite in KwaZulu-Natal is the *Plasmodium falciparum* which is transmitted primarily by *Anopheles arabiensis*, which is the main vector of malaria in South Africa. *Anopheles funestus* and *Anopheles merus* may act as secondary vectors. A study in KwaZulu-Natal identified a potential new vector, *Anopheles vaneedeni,* which tested positive for *P. falciparum* sporozoites [5]. Following the 1999/2000 malaria outbreak, South Africa and in particular KwaZulu-Natal has seen a sustained decrease in malaria prevalence through various control strategies [6]. The government implemented a two-pronged intervention strategy across all affected provinces. This approach targeted the vector using indoor residual spraying (IRS) and timely issuing of artemisinin-based combination therapy (ACT) to control the parasite [7]. South Africa is targeting elimination by 2025 with KwaZulu-Natal poised for elimination by 2023. eSwatini on the northern side of KwaZulu-Natal is also targeting elimination by 2020, but the north-eastern neighbour, Mozambique who contributed to 5% of the worlds malaria cases in 2017 is still in the control phase [1]. The Malaria Elimination Strategic Plan of South Africa [8] highlights the main thematic areas as being programme management, case management, vector control, advocacy, surveillance and operational research. Limpopo province is still in the control phase with Mpumalanga province being in the early stages of elimination. KwaZulu-Natal is furthest along in implementing the elimination agenda and we focus the changes in this province over the past 10 years. The foundation of malaria prevention in KwaZulu-Natal is vector control. The KwaZulu-Natal Malaria Programme focuses on IRS and foci clearing as well as case management. An obstacle facing the KwaZulu-Natal malaria elimination programme is the increased and frequent population movement across South Africa’s borders. The South African national Department of Health has implemented cross-border strategies to strengthen prevention and provide treatment services in the exporting areas of neighbouring countries.

The objective of this study was to investigate the changes in epidemiology over the past decade (2008–2018) with a view to determining how close to elimination the province really is. This paper draws attention to KwaZulu-Natal and its drive towards elimination. It focuses on the technical and operational strategies as well as the challenges faced by the province in achieving its elimination targets. This information can then contribute towards the South African 2025 elimination strategy.

Since there is some literature on the progress of KwaZulu-Natal towards elimination, key information will be presented namely, local and imported malaria cases and deaths, cases in children < 5 years of age and pregnant women, IRS coverage and the population protected. These are the main indicators that highlight the progress towards elimination in KwaZulu-Natal.

## Methods

The data used in this study was drawn from the KwaZulu-Natal Malaria Information System (MIS). The KwaZulu-Natal malaria surveillance system is based on national guidelines [9].

### Morbidity and mortality data

Malaria cases that are diagnosed at health facilities are recorded in a health facility case register and communicated telephonically to the district health office. Individual case records from malaria surveillance conducting active case detection are also routinely entered onto malaria notification forms. These forms are submitted on a weekly basis to provincial malaria control programmes (MCP). Here, individual case data including patient details, symptoms, diagnosis, microscopy and/or rapid diagnostic test (RDT) results, treatment administered, referrals information, the locality the patient resides in and the reporting health facility’s name are entered onto a computerized malaria information system (MIS). Malaria surveillance agents are provided with case reports generated by the MIS for follow-up and investigation. Upon conclusion of case investigations, case follow-up forms are returned to the provincial MCP where any new information (such as treatment outcome and potential source of infection) is entered into the MIS. The MIS allows for data entry at the individual patient level or as spatial (geographically) and temporal (over time) aggregates. The data required for the study was limited to the variables identified, temporally for the decade starting from 2008 to 2018.

The case data is usually reported from a health facility but malaria surveillance agents also report on case data as they are engaged in active case detection when conducting monthly household visits in the malaria affected parts of the province. Diagnosis is done using rapid diagnostic tests at all levels of health care, but blood smears are often examined at referral hospitals. Diagnosis is conducted by trained malaria control staff but administration of artemether–lumefantrine is undertaken at health facilities by health professionals. Positive cases diagnosed by malaria surveillance agents are referred to the nearest health facility for treatment. During case investigation or foci clearing, nurses are part of the process and can treat people in the communities. All cases of malaria are investigated within 24 h to determine whether these are local or imported cases. Imported cases are defined as those cases, which were contracted outside the border of the country, as indicated by their travel history. To identify and prevent the spread of disease, screening at identified border entry points are conducted routinely.

Although all cases are recorded, the number of cases of pregnant women and children under 5 are regarded as an important indicator to track trends in low transmission settings [10]. Since pregnant women and children under 5 years are particularly vulnerable to infection, major changes in disease transmission would be more apparent in this population [10]. While all case data is presented as absolute numbers, incidence data may provide a more meaningful interpretation of the data. However, incidence data could not be calculated for the study area as the malaria distribution does not include full municipalities and districts and calculating population by extrapolating from census data would have incorrect data since only segments of the municipalities are affected by malaria.

### Indoor residual spraying

Indoor residual spraying (IRS) which is the main strategy used for vector control is undertaken in the three malarious provinces of South Africa. IRS operations use insecticides such as dichloro-diphenyl-trichloro-ethane (DDT), carbamates or pyrethroids. Homesteads that form part of the active malaria control and surveillance programme are sprayed by trained and qualified spray officers. Records of each spray session are captured onto a spray card which are then submitted to the IRS team leader for verification of data quality and completeness. The card is thereafter sent to the provincial MCP offices where the data is entered into a customized spray information system (SIS). The SIS system allows for data entry, querying and reporting. The system also allows managers to monitor various aspects of the spray process such as progress, coverage, performance of individual spray officers, insecticide usage and application rates via predefined reports. The SIS was implemented country-wide in 2005 and thus data is only available from that time. Previously IRS data was collected in different forms across the different provinces. The only variables drawn from this database was percent coverage and population protected from 2001 to 2018. Percentage spray coverage refers to the proportion of existing structures sprayed while population protected refers to the actual number of people living in the sprayed structures.

## Results

**Fig. 1:**
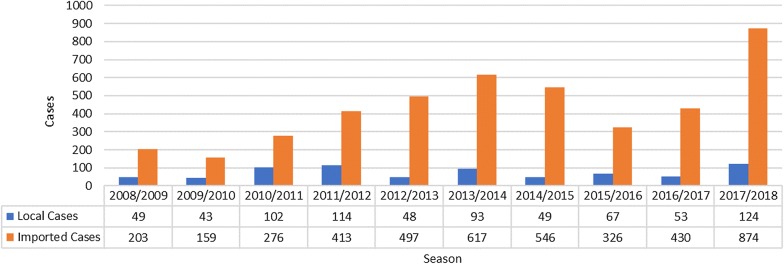
Local and imported cases in the province

Although KwaZulu-Natal experienced a severe epidemic in the 1999–2000 malaria season, the number of cases has decreased dramatically in the last 20 years but there has been an increase in the number of cases over the past decade (Fig. 1). The increase in the number of cases is cause for concern since KwaZulu-Natal was the province with the highest burden of disease prior to 2000. Through the implementation of effective parasite and vector control strategies, the cases in KwaZulu-Natal has decreased to those shown in Fig. 1.

However, this decrease from the 2013–2014 to the 2015–2016 season reversed during the 2017/2018 malaria transmission season with an upward trend in the number of cases reported. In KwaZulu-Natal, the indigenous cases make up a small proportion of the total number of cases, with most of the cases being recorded as being imported from outside the borders of the country (Fig. 1) and it is these imported cases that also cause secondary cases within the province. This is evidenced by the fact that as imported cases have increased, so have the number of local cases even though the reported increase in the number of local cases have been slight in the last three malaria seasons.

**Fig. 2:**
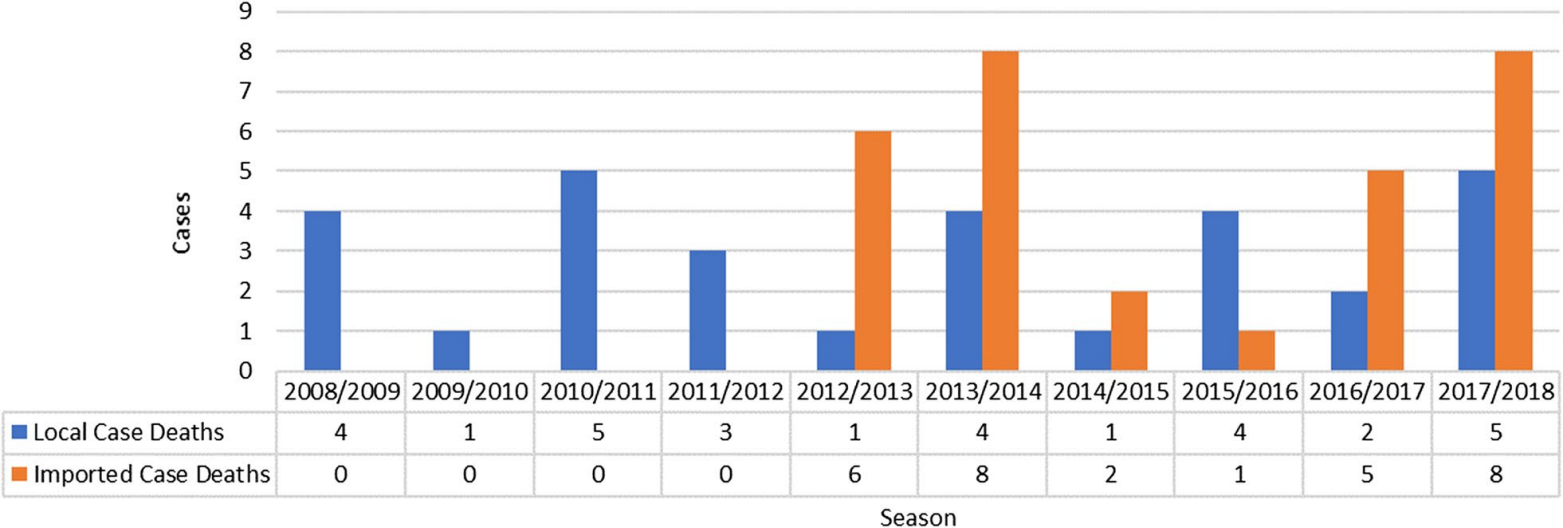
Deaths due to local and imported cases

The number of deaths due to malaria has also increased during the past ten seasons with deaths due to imported malaria increasing in importance (Fig. 2). The number of deaths due to local malaria has also increased fivefold when comparing the data for 2017/2018 with that of 2011/2012 malaria seasons. The number of deaths due to imported malaria has also increased from zero in 2011/2012 to 8 in 2017/2018. During the early part of the decade, no deaths were recorded from imported cases. Over the past 3 years especially, the number of deaths due to imported malaria has increased markedly and the number of deaths due to local malaria increased by 25% when compared to the 2016/2017 season. Although the numbers are low, the implications of increasing deaths hamper the elimination agenda.

### Malaria in vulnerable groups

**Fig. 3:**
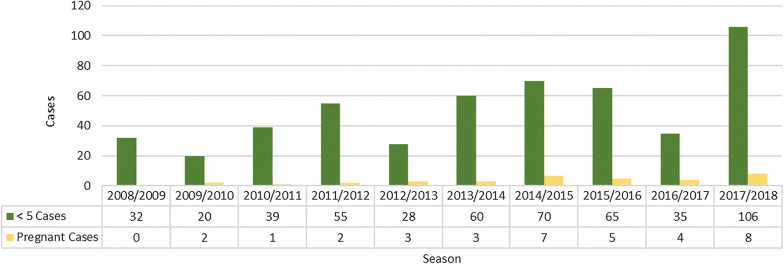
Malaria in vulnerable groups

Globally, children under the age of 5 years and pregnant women are considered to be the main groups vulnerable to malaria. Children under 5 years of age contribute approximately 10% to the burden of disease in KwaZulu-Natal (Fig. 3), when examining the local cases. The number of local pregnant women also contribute very minimally to this case load with this vulnerable group accounting for less than 1% of the number of cases being reported from this endemic province. The data for local cases once again highlights the fact that the main transmission occurs in adults who spend more time outdoors away from the protection afforded by IRS.

### Indoor residual spraying

**Fig. 4:**
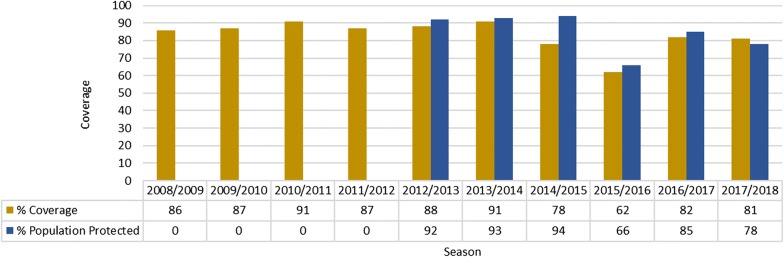
Percentage household sprayed and population protected (0 = no data available)

Since IRS is currently the only vector control intervention employed in the province it is essential to determine how spray coverage affects the burden of disease. In the early part of this decade coverage with IRS was maintained at over 85% (Fig. 4).

The population protected was also over 85%. An anomaly occurs in 2014 when the spray coverage is below 80% but the population protected is higher than 90% since the malaria control programmes targeted the high risk, high population density areas. Even though a smaller proportion of houses were sprayed, higher numbers of people lived in these sprayed structures. The IRS targets were not achieved in the 2015/2016 season with very low IRS coverage and consequently low population protected. This also saw an increase in the number of malaria cases reported from the province. In the remaining years of the reporting period, IRS coverage has never reached the levels reached in the early years of this decade and consequently there has been an increase in number of cases, especially imported cases (Fig. 1). Further to the information provided in Fig. 4, during the 2015–2016, 2016–2017 and 2017–2018 malaria seasons, 233,798, 329,810 and 157,462 structures respectively were targeted for spraying but only 143,801, 268,936 and 126,801 of these structures were sprayed.

Information on population protected with IRS was not recorded prior to the 2012–2013 season but extrapolating from available data, it was indicative that at least 80% of the population was protected by IRS in the early years of the decade.

## Discussion

In comparison with the other malaria endemic provinces in the country, KwaZulu-Natal will attain malaria elimination by 2023 [11]. Although very low number of malaria cases and deaths are being reported from the province, there has been a marginal increase in both these variables over the past three malaria transmission seasons. As the province teethers on the brink of elimination, imported malaria is becoming more of a problem and this issue needs to be addressed before KwaZulu-Natal can achieve the desired goal by 2023. Also, adults are more susceptible to transmission than young children and other special groups as the case numbers in these vulnerable groups are very low. The preventative measures such as IRS is not being implemented effectively (< 80% spray coverage), contributing to an increase of cases. This increase may partially be a result of secondary transmission from imported malaria cases. Since there is limited screening for malaria at border crossings, infected individuals can freely move into the province from high burden areas such as from neighbouring Mozambique. Also, as a consequence of the low number of cases being identified in the country, implementing enhanced border-screening would not be any more effective and would be a waste of resources.

A recent review of the KwaZulu-Natal Malaria Control Programme showed that there were gaps in critical areas that could hamper the goal of elimination [12]. Some of the low spray coverage can be blamed on an inefficient procurement system since ordering of insecticides was delayed. Also, there was no permanently employed manager appointed to oversee the programme implementation and this could have contributed to poor planning of the IRS campaigns which would have led to the low spray coverage and thus the increase in number of reported cases. Another factor which may have influenced the spray coverage is the change in housing type from the more traditional mud and daub to cement and painted structures. Spray coverage may also have decreased by people refusing to have their modern homes sprayed with an insecticide.

One of the factors contributing to residual malaria is the unknown role of secondary vectors. Traditional malaria control tools have targeted only *Anopheles gambiae s.s.*, *An. arabiensis* and *An. funestus*. Targeting control measures at the three species has resulted in huge reductions in the burden of disease. However, in recent times new vectors have been identified [5]. It may also be that these new vectors that feed and rest outdoors contribute to very low levels of transmission. Since the vector control tools currently employed have failed to control residual transmission, several strategies are being rolled out to assist with the less effective vector control tools. According to Killeen [13], the issue of residual malaria can be addressed by novel or improved vector control strategies that address residual transmission. Such strategies enhance control of vectors that enter houses to feed and/or rest by killing, repelling or excluding them. Vectors have adapted their behaviour to feeding earlier and outdoors providing another obstacle to elimination.

Notwithstanding the above factors, KwaZulu-Natal is further ahead than the other endemic provinces in its attainment of elimination. eSwatini was poised to achieve elimination in 2015 but this would now most likely occur at the same time as KwaZulu-Natal in the early 2020s [14]. The implementation of cross-border malaria control creates an opportunity for KwaZulu-Natal to tackle the sources of imported malaria in neighbouring countries, especially Mozambique [15, 16]. South Africa has recently sourced funding to target the sources of KwaZulu-Natal’s imported cases in Gaza and Inhambane provinces of Mozambique. Even within the Elimination 8 countries, South Africa will be among the first to eliminate malaria in southern Africa, but KwaZulu-Natal is on track for sub-national certification in 2023 [11]. A comparison of the malaria morbidity and mortality of the SADC region with the E8 region shows that the disease burden in the eight southern-most countries on the continent is very low when compared to their northern neighbours and the countries on the fringes of malaria distribution will eliminate malaria first [17].

Like all countries targeting elimination, residual malaria is challenging current efforts to reach elimination. Low levels of transmission are preventing the province from attaining elimination. Since mosquito vectors can adapt their behaviour [18], the challenges facing the traditional methods of vector control is that they are no longer as effective since mosquitoes are now feeding earlier in the day and also feeding outdoors. This behaviour modification renders the use of IRS and long-lasting insecticide-treated net as obsolete and novel methods need to be developed such as outdoor control [19]. Also, greater attention is now being given to larval control thorough the use of chemicals as well as environmental modification to decrease the number of viable vector breeding sites. Moreover, more expensive methods such as house modification and the use of screens should be considered where the efficacy of insecticides is waning to the commonly used chemicals. However, the greatest challenge would be to prevent secondary cases that result from imported cases through real time notification and case investigation. Although, initiatives such as the Lubombo Spatial Development Initiative and MoSaSwa (Mozambique, South Africa, eSwatini) have made significant contributions to achieving the elimination goal [15, 16] greater focus should be on the sources of infection that are fuelling the low transmission of malaria in the province.

One of the limitations of this study was the inability to estimate standardized incidence rates due to the distribution of the disease not covering entire municipalities and districts, the lowest level at which population data is available in South Africa.

## Conclusion

Even though there are major impediments to achieving elimination, the changing epidemiology suggests that major strides have been made in the past 10 years and KwaZulu-Natal is on track to achieving this milestone by 2023. The application of indoor residual strategy needs to be improved both in terms of structures sprayed and population protected. More attention needs to be paid to mobile and migrant populations, especially asymptomatic individuals, to reach the elimination goal and to prevent reintroduction of malaria to areas that are malaria free in the province.

## Data Availability

All datasets used and/or analysed during the current study are available from the corresponding author on reasonable request.

## Abbreviations

ACT: artemisinin-based combination therapy
DDT: dichloro-diphenyl-trichloro-ethane
IRS: indoor residual spray
MIS: malaria information system
MCP: malaria control programme
RDT: rapid diagnostic test
SIS: spray information system
